# Laboratory and Field-Based Evaluation of Short-Term Effort with Maximal Intensity in Individuals with Intellectual Disabilities

**DOI:** 10.1515/hukin-2015-0092

**Published:** 2015-01-12

**Authors:** Judit Lencse-Mucha, Bartosz Molik, Jolanta Marszałek, Kalina Kaźmierska-Kowalewska, Anna Ogonowska-Słodownik

**Affiliations:** 1 Department of Sport for People with Disabilities, Faculty of Physiotherapy, University of Physical Education in Warsaw, Poland; 2 Department of Theory and Methodology in Teaching Movement of People with Disabilities Faculty of Physiotherapy, University of Physical Education in Warsaw, Poland; 3 Department of Adapted Physical Activity Faculty of Physiotherapy, University of Physical Education in Warsaw, Poland

**Keywords:** anaerobic performance, physical fitness, intellectual disability, Wingate test

## Abstract

Results of previous studies have not indicated clearly which tests should be used to assess short-term efforts of people with intellectual disabilities. Thus, the aim of the present study was to evaluate laboratory and field-based tests of short-term effort with maximal intensity of subjects with intellectual disabilities. Twenty four people with intellectual disability, who trained soccer, participated in this study. The 30 s Wingate test and additionally an 8 s test with maximum intensity were performed on a bicycle ergometer. The fatigue index, maximal and mean power, relative maximal and relative mean power were measured. Overall, nine field-based tests were conducted: 5, 10 and 20 m sprints, a 20 m shuttle run, a seated medicine ball throw, a bent arm hang test, a standing broad jump, sit-ups and a hand grip test. The reliability of the 30 s and 8 s Wingate tests for subjects with intellectual disability was confirmed. Significant correlation was observed for mean power between the 30 s and 8 s tests on the bicycle ergometer at a moderate level (r >0.4). Moreover, significant correlations were indicated between the results of laboratory tests and field tests, such as the 20 m sprint, the 20 m shuttle run, the standing long jump and the medicine ball throw. The strongest correlation was in the medicine ball throw. The 30 s Wingate test is a reliable test assessing maximal effort in subjects with intellectual disability. The results of this research confirmed that the 8 s test on a bicycle ergometer had a moderate correlation with the 30 s Wingate test in this population, thus, this comparison needs further investigation to examine alternativeness of the 8 s to 30 s Wingate tests. The non-laboratory tests could be used to indirectly assess performance in short-term efforts with maximal intensity.

## Introduction

Laboratory aerobic endurance tests have been frequently used and described by researchers (Fernhall and Pitetti, 2001; Beynard at al., 2008; Pitetti at al., 2012). The level of peak aerobic power (VO2peak) has been well established in people with intellectual disability (ID) (Baynard et al., 2008). Previous studies have demonstrated that young subjects with ID had lower cardiovascular performance (Yarmer et al., 2000; Pitetti et al., 2012) and a higher level of body fat than their peers without ID (Wondra et al., 2000; Frey, 2004). People with ID also presented a lower level of physical activity compared to their peers without ID. Many studies demonstrated data concerning low physical fitness (Pittetti et al., 2012; Fernhall and Pitetti, 2001; Guerra et al., 2000; Jin-Ding et al., 2010). Low muscle power in individuals with ID was also showed (Pitetti and Boneh, 1995; Guerra et al., 2000; Fernhall, 1996; Horvat et al., 1999). Blomqvist et al. (2013) reported poor balance in people with this disability. Only the study of Chia et al. (2002) examined short time performance with maximal intensity in subjects with ID.

Intellectual disability is not a contraindication for high intensity anaerobic performance. People with ID participate in anaerobic-type training in the Special Olympics e.g. gymnastics, powerlifting, a 100 m run, basketball and soccer. Therefore, studies evaluating anaerobic capacity of people with ID could give a more complete picture of physical capabilities of this population.

The Wingate Anaerobic Test (WAnT) is the most popular high intensity test (Inbar et al., 1996). This test is used to determine peak power (PP), mean power (MP) and the fatique index (FI) while pedalling at maximum speed for 30 s on a cycle or arm crank ergometer (Chia et al., 1997). The WAnT is a test commonly used in rehabilitation and sports for subjects with disabilities. The reliability of this test was validated in many groups i.e. the able-bodied (Inbar et al., 1996), people with physical disabilities (Hutzler et al., 2000; Jacobs et al., 2003; Molik et al., 2005), wheelchair basketball players (Goosey-Tolfrey, 2005; Molik, 2013; Vanlandewijck et al., 1999), individuals with para- and quadriplegia (Jacobs et al., 2003), children, both healthy and with physical disabilities (Tirosh et al., 1990), children with juvenile idiopathic arthritis (Stephens et al., 2007).

Chia et al. (2002) evaluated reliability and variability of the 30 s Wingate test in a group of boys with intellectual disability and also compared WAnT performance of boys with and without ID. High reliability was noticed for MP and PP at 0.95 and 0.93, respectively, for boys with ID and 0.97 and 0.90 for peers without ID. That study showed that boys with ID had comparable levels of reliability in the WAnT to boys without ID, however, the data was more variable in the disabled group. The authors were not certain whether the variability in the WAnT power test was due to a lack of motivation on the part of the boys with ID, or it was the reason of high intensity exercise outcomes in boys with ID. It has been reported that WAnT performance in special populations is not only inferior to those of normal populations, but also more variable (Inbar et al., 1996).

Results of previous studies did not clearly indicate which laboratory and non-laboratory (field) tests should be used to assess short-term efforts of people with intellectual disabilities. The Wingate test was very rarely used in studies in people with ID. Thus, the aim of the present study was to evaluate laboratory and field-based tests of short time effort with maximal intensity in people with intellectual disability.

## Material and Methods

### Participants

Twenty-four subjects with ID aged 18–30 years volunteered to participate in this study. Adult Special Olympics athletes composed the research sample. All subjects agreed to participate in the study by signing the consent form. The study was conducted during the Special Olympics training camp in the Olympic Sports Centre in Spała (Poland), between the 9^th^ and 15^th^ of May, 2014. All study participants were classified as having moderate ID. Subjects with Down Syndrome did not participate in the study. The average age, body height, body mass, and BMI were: 22.46 years (SD=3.84), 171.08 cm (SD=8.57), 71.54 kg (SD=13.09) and 24.39 kg/m^2^ (SD=3.87), respectively.

The participants were informed about the possibility to withdraw from the research at any time. None of the subjects resigned from participation. Inclusion criteria for the subjects to participate were as follows: intellectual disability without any other dysfunction, without any cardiorespiratory or pulmonary diseases.

### Measures

A weight scale (kg) and a stadiometer (cm) were used for anthropometric measurements. The laboratory tests consisted of the 8 s and 30 s WAnT (Inbar et al., 1996; Chia et al., 2002; Guerra, 2009). The tests were conducted on a Monark cycle ergometer (Ergomedic 874E) interfaced with a computer. For the selection of the most optimal resistance to use during the basic test (30 s), pre-tests were performed for all participants. During the pre-tests, peak power (PP) was monitored and the time at which it was achieved. The resistance load, with which the best values of PP were registered, was applied during the 30 s WAnT.

The field-based tests were conducted on a synthetic track. Most of the non-laboratory tests derived from selected tests from the Eurofit test battery or the International Physical Fitness Test. The participants performed 9 tests: 5, 10 and 20 m sprints, a 20 m shuttle run in 30 s, sit-ups in 30 s, a seated medicine ball throw (3 kg) with both hands from the chest, bent arm hang on a bar, a standing broad jump using two foot take-off and landing and a hand grip test (kg). Each subject completed the tests individually. The examiner verbally encouraged participants during tests, but was not allowed to help in any other way.

### Procedures

The subjects participated in four sessions of measurement. The first one was the laboratory testing, i.e. anthropometric measurements and pre-tests. As a pre-test, 8 s cycling with maximal speed was used and it was conducted in groups of five people. After a warm-up, i.e. 2 min of pedalling between 50–60 rpm, they stopped and started the pre-test with 3% of their body mass. The load increased by 1% in each following trial. After this short exercise, the participant rested until his heart rate (HR) decreased to the resting level, and then he performed a trial with higher resistance. The participants continued performing 8 s tests until they could not achieve higher PP. If a subject reached 0.74 N per kilogram of body mass, he would not continue the pre-tests. Each of them was allowed a maximum of five trials. The participants were verbally encouraged throughout the tests. During the 8 s tests the subjects went through the familiarization process. These trials were helpful in teaching the participants the correct execution of the exercise. After establishing the most adequate load for each of the participants, they repeated the 8 s test the second time (with the same load).

In the second session, the standardized 30 s WAnT was performed (Inbar et al., 1996 ). The session consisted of a warm-up (pedalling for 5 min at 60 rpm and load 50W), 2–3 min of rest, the 30 s WAnT and a cool down (pedalling for 2 min). The test was started after a countdown of “3, 2, 1, go”, and then, the participant began pedalling on the ergometer at maximum speed and continued for 30 s. During the WAnT, mean power (MP), relative mean power (rMP – power per kilogram of body mass), peak power (PP), relative peak power (rPP – power per kilogram of body mass), and the fatigue index (FI - determined on the basis of power decline) were measured.

The third session was conducted one day after the second day of testing. The participants repeated the 30 s WAnT, step by step like in the previous session.

The fourth session included the field-based measurements to assess participants’ physical fitness and abilities. The tests consisted of a handgrip test (dynamometer), track events (5, 10 and 20 m sprints), a 20 m shuttle run test, a standing broad jump, sit-ups in 30 s, a modified seated medicine ball throw (3 kg), and the bent arm hang test.

Each subject completed all tests. The participants of the study performed the tests under similar conditions, and were verbally encouraged during tests. The same examiner conducted all tests for all subjects.

### Statistical analysis

All statistical analyses were performed using IBM SPSS Statistics 21. The Pearson correlation coefficient was used to assess the level of reliability of laboratory anaerobic tests, and to determine the relationships between anaerobic performance parameters and all field tests. An alpha value of *p* < .05 was considered statistically significant.

## Results

The mean values, standard deviations, minimum and maximum values for all the test variables are presented in Table 1.

The reliability of the 30 s and 8 s WAnT was confirmed. The results for the reliability of two trials of the 30 s Wingate test showed a significant correlation level between the tests (MP .97; rMP .91; PP .97; rPP .88; FI .88). In the 8 s Wingate test the correlations were lower in comparison to the 30 s WAnT, but also high (MP .68; rMP .66; PP .75; rPP .76). The results revealed that subjects with ID reached high reliability in anaerobic tests.

The correlations between the 30 s and 8 s Wingate tests and all field tests are summarized in Table 2. The 30 s WAnT correlated with the handgrip test (MP, rMP, PP), the 20 m shuttle run (rPM, rPP), the medicine ball throw (MP, rMP, PP, rPP) and bent arm hang tests (MP, rMP). The 8 s WAnT correlated with the 20 m shuttle run (rMP, rPP), the broad jump (all parameters) and sit-up tests (rMP). Strongest correlation was indicated between MP and PP measured in the 30 s WAnT and the medicine ball throw test (r = .642 and r = .659, respectively). The strongest correlation between MP and PP measured in the 8 s WAnT was found for the medicine ball throw test (r = .655 and r = .668, respectively).

Table 3 demonstrates the correlations between the two short-term exercise tests (30 s and 8 s) in measured values. The correlations between the tests are statistically significant in mean power, relative mean power, maximum power and maximum relative power.

## Discussion

All participants of this study had moderate intellectual disability (Luckasson and Reeve, 2001). Some studies demonstrated that people with ID had higher levels of body fat than their peers without ID (Yamaki and Taylor, 2005). In the present study, subjects with ID had a BMI value of 22.46, i.e. in normal weight status. Freedman et al. (2010) reported that the BMI of able-bodied men (mean age=30, n=1427) was 26.5. Subjects of this study, who were males with ID, were leaner than their peers without disability in the Freedman et al.’s (2010) study.

The aim of the study was to evaluate laboratory and field-based tests of short-term effort with maximal intensity in subjects with intellectual disabilities. The reliability coefficients in the 30 s WAnT of the active men with ID were high for MP, rMP, PP and rPP at 0.97, 0.91, 0.97 and 0.88, respectively, and comparable to the results of young men without ID (Inbar et al., 1996) as well as adolescents with ID (Chia et al., 2002). The level of reliability of the 8 s WAnT was lower in comparison to the 30 s WAnT. Results confirmed reliability of both tests, however, further analyzes of shorter tests based on the phosphagen system are recommended.

In this study, the reliability of the fatigue index (FI) in the 30 s test-retest was at a high level (p< .05, FI 0.88). In other research the authors indicated a low level in relative and absolute reliability of the FI for the 20 and 30 s test duration in a group without disabilities (Attia et al., 2014). The reliability of this parameter requires further studies to confirm the results.

However, the reliability of the 30 s Wingate test in people with intellectual disability with Down syndrome is questionable (Guerra et al., 2009). The research showed statistically significant difference only in PP values (0.93, *p*<.05) and the authors suggested that more practice or more tests might be needed to receive reliable results in this group of disability. Perhaps if the testing time was shortened, the reliability coefficient would be higher. Reliability of the 20 s Wingate test was determined for the abled-bodied (Attia et al., 2014) and disabled group (Dallmeier et al., 2012). Attia et al. (2014) demonstrated that the 20 s WAnT was a reliable tool for evaluation of anaerobic performance of legs in male team sport athletes, while Dallmeier et al. (2012) showed that the test-retest reliability of mean power output was excellent (0.96, *p*<.05) and the peak power output was lower, but still reliable (0.85, *p*<.05) in children with cerebral palsy.

In the current study throughout every trial examiner verbally encouraged participants to put maximal effort into the 30 s test. High correlation coefficients in the 30 s WAnT indicated no motivation problem to perform the task by subjects with ID. However, in literature behavioral difficulties of people with ID were noted (Cromarck et al., 2000). More studies concerning this issue are necessary to solve the problem.

Significant relationships between the values of Wingate tests and filed-based tests indicate that field tests are a good tool for Special Olympics coaches or occupational therapists to evaluate non-laboratory anaerobic performance of people with ID. The strongest correlation was observed in the seated medicine ball throw from the chest and values of MP, rMP, PP, rPP in 8 s and 30 s tests; other tests had lower but significant correlations in the hand grip test and MP30, rMP30, PP30; the 5 m sprint and FI30; the 20 m shuttle run and rMP30, rPP30, rMP8, rPP8; sit-ups and rPM8; the bent arm hang test and MP30, rMP30. The results indicated that the medicine ball throw correlated most strongly with laboratory tests. Other research presented similar results (Molik et al., 2013), where the strongest correlation was revealed between MP, PP, and the field test measuring the two-handed chest pass of female wheelchair basketball players. Vanlandewijck et al. (1999) demonstrated a high correlation between the shuttle run and the anaerobic laboratory test (arm cranking) in wheelchair basketball players. Among the other field tests, the shuttle run test is a reliable and valid tool for examining anaerobic capacity and skill proficiency assessment in wheelchair basketball players. No information about this issue in people with intellectual disability was found in the literature.

The outcomes of this study did not confirm the assumption that the 8 s and 30 s WAnT would be highly correlated. Although the correlations were significant, the values were on a moderate level i.e. 0.49, 0.47, 0.44 and 0.44 for MP, rMP, PP and rPP, respectively. The results showed that the 8 s test could be used as an alternative to the 30 s Wingate test. Although further investigations are needed to confirm or discard this finding. Similar analyzes were performed in able-bodied competitive athletes. Zając et al. (1999) reported that the 10 s version of the Wingate test can be used for evaluation of maximal power. Our analyzes confirmed the results of previous studies. Shorter tests (8 or 10 s) are based on the phosphagen system and induce maximal power in people with and without disabilities. The longer (30 s) test is a good predictor for evaluation of the effectiveness of the glycolic system. Mean power is a good indicator of the effectiveness of submaximal efforts for prolonged time i.e. 15–20 s (Zając et al., 1999). Anyway, Zając et al. (2013) reported higher results of the 10 s WAnT in comparison to the 30 s WAnT. We are not able to confirm previous investigations’ findings due to differences in the 8 s WAnT between first and second tests. However, in the second 8 s WAnT individuals achieved better results of peak power and mean power in comparison to the 30 s WAnT.

Further research regarding this matter is recommended primarily in untrained subjects with intellectual disability and with Down syndrome where the results are not clear. It seems justified to shorten the time of the WAnT to 15, 10 or 8 s to achieve greater reliability in subjects with ID.

## Conclusion

The two tests of 8 and 30 s can be used to evaluate short-term efforts with maximum intensity in people with intellectual disabilities (without Down syndrome). The medicine ball throw seems to be the best test to indirectly (non-laboratory) evaluate muscular power of subjects with intellectual disability.

## Figures and Tables

**Table 1:** Values of the 30 s and 8 s Wingate tests and field-based tests of subjects with intellectual disability

VARIABLE		MEAN	SD	MIN	MAX
Filed Tests	Handgrip	78.05	20.55	48.00	131.00
	5 m sprint	1.70	0.23	1.24	2.27
	10 m sprint	2.59	0.26	2.20	3.30
	20 m sprint	4.25	0.41	3.70	5.20
	20 m shuttle run	125.48	10.34	106.00	140.00
	Broad jump	1.74	0.31	1.17	2.35
	Ball throw	5.02	0.72	4.00	6.50
	Sit-ups	19.21	4.62	10.00	29.00
	Bent arm hang	27.45	17.38	7.98	72.12
1st – 30 s WAnT	MP30_1	458.14	121.39	286.90	868.20
	rMP30_1	6.41	1.06	4.43	8.28
	PP30_1	606.88	181.26	330.10	1229.20
	rPP30_1	8.45	1.58	5.00	11.38
	FI30_1	18.17	12.74	0.06	37.18
2nd – 30 s WAnT	MP30_2	445.75	117.82	289.50	870.50
	rMP30_2	6.21	1.11	3.90	8.32
	PP30_2	585.13	180.49	329.10	1234.10
	rPP30_2	8.07	1.70	3.46	11.21
	FI30_2	18.22	12.02	0.10	35.26
1st – 8 s WAnT	MP8_1	412.13	150.74	142.20	680.39
	rMP8_1	5.78	1.95	2.49	8.72
	PP8_1	539.77	188.30	210.60	872.80
	rPP8_1	7.58	2.44	3.69	11.32
2nd – 8 s WAnT	MP8_2	483.67	141.42	220.00	784.80
	rMP8_2	6.70	1.48	3.86	9.33
	PP8_2	612,72	170.08	320.70	919.10
	rPP8_2	8.50	1.79	5.63	11.56

The first trial of the 30 s Wingate test: MP30_1 - mean power, rMP30_1 - relative mean power, PP30_1 - peak power, rPP30_1 - relative peak power, FI30_1 - fatigue index. The first trial of the 8 s Wingate test: MP8_1 - mean power, rMP8_1 - relative mean power, PP8_1 - peak power and rPP8_1 - relative peak power. The second trial of the 30 s Wingate test: MP30_2 - mean power, rMP30_2 - elative mean power, PP30_2 - peak power, rPP30_2 - relative peak power, FI30_2 - fatigue index. The second trial of the 8 s Wingate test: MP8_2 - mean power, rMP8_2 - relative mean power, PP8_2 - peak power, rPP8_2 - relative peak power.

**Table 2:** Correlations between the 30 s and 8 s tests and all field tests

		MP30	rMP30	PP30	rPP30	FI30	MP8	rMP8	PP8	rPP8
		
Handgrip	R	.618**	.481*	.545*	.420	.148	.277	.070	.255	.035
	P	.005	.037	.016	.074	.544	.252	.775	.293	.885
5 m sprint	R	−.167	−.325	−.173	−.327	−.407*	−.086	−.143	−.082	−.142
	P	.435	.121	.419	.119	.048	.690	.504	.703	.507
10 m sprint	R	−.213	−.376	−.183	−.330	−.097	−.324	−.383	−.327	−.380
	P	.317	.070	.391	.115	.652	.122	.065	.119	.067
20 m sprint	R	−.341	−.302	−.292	−.248	−.001	−.405	−.345	−.380	−.305
	P	.153	.209	.226	.305	.997	.085	.148	.108	.204
20 m shuttle run	R	.159	.587**	.156	.547**	.026	.409	.597**	.409	.599**
	P	.469	.003	.477	.007	.907	.052	.003	.052	.003
Broad jump	R	.134	.305	.159	.345	.116	.539**	.601**	.565**	.626**
	P	.533	.148	.459	.099	.589	.007	.002	.004	.001
Ball throw	R	.642**	.485*	.659**	.580**	.096	.655**	.475*	.668**	490*
	P	.001	.022	.001	.005	.670	.001	.025	.001	.021
Sit-ups	R	.018	.369	−.043	.247	.001	.231	.416*	.166	.344
	P	.935	.076	.842	.245	.997	.277	.043	.439	.100
Bent arm hang	R	.566*	.574*	.400	.398	−.012	.021	−.004	−.064	−.098
	P	018	016	.111	.114	.962	.938	.988	.808	708

* p < 0.05;

** p < 0,01;

MP30-mean power, rMP30-relative mean power, PP30-peak power, rPP30-relative peak power, FI30-fatique index for the 30 s WAnT; MP8-mean power, rMP8-relative mean power, PP8-peak power, rPP8-relative peak power for the 8 s WAnT

**Table 3:** Correlations between the 30 s and 8 s Wingate tests

		MP8	rMP8	PP8	rPP8
		
MP30	r	.486*	.217	.432*	.162
	p	.016	.309	.035	.450
rMP30	r	.371	.469*	.324	.430*
	p	.075	.021	.123	.036
PP30	r	.458*	.185	.442*	.170
	p	.025	.387	.031	.428
rPP30	r	.386	.420*	.397	.440*
	p	.062	.041	.055	.032
FI30_1	r	−.217	−.332	−.076	−.185
	p	308	.113	.725	.388

* p < 0.05;

MP30-mean power, rMP30-relative mean power, PP30-peak power, rPP30 relative peak power for the 30 s WAnT; MP8-mean power, rMP8-relaitve mean power, PP8-peak power, rPP-relative peak power for the 8 s WAnT.
